# Support for mask use as a COVID-19 public health measure among a large sample of Canadian secondary school students

**DOI:** 10.1186/s12889-022-14011-0

**Published:** 2022-08-22

**Authors:** Karen A. Patte, Terrance J. Wade, Adam J. MacNeil, Richard E. Bélanger, Markus J. Duncan, Negin Riazi, Scott T. Leatherdale

**Affiliations:** 1 Department of Health Sciences, Faculty of Applied Health Sciences, Niagara Region, Brock University, 1812 Sir Isaac Brock Way, St. Catharines, ON L2S 3A1 Canada; 2 Projet COMPASS-Québec, VITAM – Centre de recherche en santé durable de l’Université Laval, 2480 chemin de la Canardière, Quebec City, QC G1J 2G1 Canada; 3 Departement of Pediatrics, Faculty of Medecine, Université Laval, Ferdinand Vandry Pavillon, 1050 Avenue de la Médecine, Quebec City, QC G1V 0A6 Canada; 4 School of Public Health Sciences, University of Waterloo, 200 University Ave., Waterloo, ON N2L 3G1 Canada

**Keywords:** COVID-19, Pandemic, Masks, School, Policy, Adolescent, Youth

## Abstract

**Background:**

Youth voice has been largely absent from deliberations regarding public health measures intended to prevent SARS-CoV-2 transmission, despite being one of the populations most impacted by school-based policies. To inform public health strategies and messages, we examined the level of student support of mask use in public spaces and school mask requirements, as well as factors associated with students’ perspectives.

**Methods:**

We used cross-sectional survey data from 42,767 adolescents attending 133 Canadian secondary schools that participated in the COMPASS study during the 2020/2021 school year. Multinomial regression models assessed support for i) wearing a mask in indoor public spaces and ii) schools requiring students to wear masks, in association with COVID-19 knowledge, concerns, and perceived risk.

**Results:**

Wearing masks in indoor public spaces was supported by 81.9% of students; 8.7% were unsupportive and 9.4% were neutral/undecided. School mask requirements were supported by 67.8%, with 23.1% neutral and 9.1% unsupportive. More females supported mask wearing in public spaces (83.9% vs. 79.1%) and school mask requirements (70.8% vs. 63.5%) than males. Students had increased odds of supporting mask use in public spaces and school mask requirements if they reported concerns about their own or their family’s health, had discussions regarding ways to prevent infection, perceived COVID-19 to be a risk to young people, and knew that signs are not always present in COVID-19 cases and that masks prevent SARS-CoV-2 transmission if someone coughs.

**Conclusions:**

During the year following the beginning of the pandemic, most students supported the required use of masks in schools and wearing masks in indoor public spaces. Improving knowledge around the effectiveness of masks appears likely to have the largest impact on mask support in adolescent populations among the factors studied.

## Background

Facial masks are shown to effectively mitigate airborne transmission of infectious severe acute respiratory syndrome coronavirus-2 (SARS-CoV-2) droplets and aerosols [1, 2]. Recommendations shifted with the fast-evolving pace of scientific discovery surrounding SARS-CoV-2 transmission, which potentially contributed to varying support for mask policies [3, 4]. In the early stages of the pandemic, handwashing and disinfecting surfaces were emphasized, with limited and conflicting communication regarding masking [5]. Leading health authorities and organizations later recommended high-quality masks as evidence of airborne transmission accumulated, a reversal of their initial stance [4, 5]. In response, many countries implemented policies requiring facial mask use in schools and other public spaces. Increased mask use followed [6]; mask mandates are positively associated with mask use in adolescents [7, 8] and schools with mask mandates had lower incidence of coronavirus disease 2019 (COVID-19) infection [9].

However, requirements around facial mask use to control the spread of COVID-19 continue to be the subject of public debate and division [4, 10]. On one hand, anti-mask protests emerged alongside mask policies [4], while on the other, the lifting of mask mandates in schools and other public spaces was deemed premature by some health authorities [11–14]. The latter pointed to increased contagiousness of (sub)variants, high levels of circulating virus, relatively lower vaccination rates in young people, and evidence of post-acute COVID-19 syndrome. Youth have been largely absent from deliberations regarding public health measures, despite being one of the populations most impacted by school-based policies and their recognized right to be involved in decisions that impact them [15]. Younger adults are less likely to support public health measures than older age groups [16, 17], but little evidence exists on adolescents’ perspectives.

Research among adults may not generalize to adolescents. During adolescence, the influence of peers and motivations to adhere to perceived social norms are heightened and greater importance is placed on peer acceptance [18]. Exercising self-determination and agency are also central to positive youth development, as a period of increasing autonomy from parent(s)/guardian(s) and identity exploration [19]. Further, early messaging suggesting COVID-19 presents little risk to younger persons may have contributed to lower risk perceptions among adolescents. Adolescents may be more exposed and susceptible to misinformation and disinformation, given their frequent social media use and lower health literacy relative to adults [20–22].

Some researchers have applied behavioural science theories to explain engagement in COVID-19 preventative actions [18, 23], which point to factors including individuals’ knowledge and beliefs regarding their susceptibility and the severity of COVID-19, perceived benefits and efficacy of engaging in the preventative action, and cues to action such as discussions with physicians, family, or other influential people. Similar factors that increase engagement in preventative behaviours may influence individual support for mask requirements. The primary objective of this study was to assess levels of support among adolescents for mask use in public spaces and for masking requirements in schools. To inform public health strategies and messages, the secondary objective was to identify factors that may drive support for mask use in adolescents, by examining associations with whether they had discussions around preventing SARS-CoV-2 infection, health concerns and perceived risk of COVID-19, and knowledge of SARS-CoV-2 transmission and mask use effectiveness.

## Methods

### Design and sample

The *Cannabis, Obesity, Mental health, Physical activity, Alcohol, Smoking, and Sedentary behaviour* (*COMPASS*) *study* is an ongoing prospective study designed to collect hierarchical survey data annually from a rolling cohort of students in grades 9 through 12 (Secondary I-V in Quebec) and their schools [24]. School boards and schools were purposefully selected based on whether they permitted active-information passive-consent parental permission protocols. All students attending participating schools and not withdrawn by their parents were eligible to participate. All participating students provided assent. Since March 2020, when schools first closed for in-person learning due to the COVID-19 public health response, COMPASS surveys were conducted online using Qualtrics XM online survey software. A survey link was emailed to all students by their schools, followed by a reminder email one week after [25]. All procedures received ethics approval from the University of Waterloo (ORE#30,118), Brock University (REB#18–099), CIUSSS de la Capitale-Nationale–Université Laval (#MP-13–2017-1264), and participating school boards. Additional details regarding COMPASS study methods can be found online [26] or in print [24].

We used cross-sectional COMPASS data from students attending 133 secondary schools (63 in Quebec, 51 Ontario, 5 Alberta, 14 in British Columbia [BC]), that participated during the 2020/2021 school year (December 2020 – May 2021). A total of 53,469 students completed the survey in 2020/2021, with an average response rate of 58.0% across schools. Students missing responses to either of the two support for mask use measures were excluded from the analysis (*n* = 8001; 7725 students were missing both mask use items). Given the small number (*n* = 287) of students that indicated that their school did not require masks, and the presence of mask policies at the time of data collections, students indicating this response were excluded. Finally, students missing predictors and covariates were removed (*n* = 2701). The final complete case sample included 42,767 participants.

### Measures

Drawing from behavioural science theories (e.g., Health Belief Model [27], Extended Parallel Process Model [EPPM] [28]), and evidence related to engagement in COVID-19 preventative measures [18, 23], we examined support for masking by perceived risk, health concerns, COVID-19 knowledge, and perceived efficacy of the preventative action. COVID-19 measures were added to the online version of the COMPASS student questionnaire [25]. Questions were adapted from a WHO questionnaire and website information and pretested by the COMPASS research team [29].

#### Mask support

Support for mask use indoors in public spaces was assessed by asking: “How true are the following statements about COVID-19 for you right now? I am supportive of wearing a mask in indoor public spaces.” Responses to a 5-point Likert scale (True, Mostly true, Neutral/I don’t know, Mostly false, False) were categorized as: True, Neutral/I don’t know, and False. Support for required mask use in schools was assessed by the question: “How supportive are you of the safety precautions your school has taken to protect against COVID-19? Requiring students to wear masks.” Provided response options included: “Supportive”, “Neutral”, “Unsupportive”, and “My school does not do this” (excluded).

#### COVID-19 discussions and mask related knowledge

Students were asked: “Since the beginning of the COVID-19 pandemic, have you done the following? I discussed measures to prevent infection with family, friends, and/or health care professionals.” Response options were dichotomized as Always/Sometimes and Never. To assess knowledge of the effectiveness of mask use, students were asked to “Please indicate which of the following statements about COVID-19 you think are true. (Mark all that apply)”, from a list that included: “When a person coughs, the use of a mask can reduce the droplet transmission of COVID-19.” In this same list, knowledge relevant to perceived need for mask use when not symptomatic, was assessed by the item: “COVID-19 is only found in individuals who show symptoms and signs of disease”.

#### Perceived risk

Perceptions of COVID-19 susceptibility and severity were assessed by asking students “How true are the following statements for you right now: I think that COVID-19 presents very little risk to young people”. Responses to a 5-point Likert scale were categorized as True, False, and Neutral/I don’t know. In addition, students were asked to “Please indicate which of the following statements about COVID-19 you think are true. (Mark all that apply)”, from a list that included: “COVID-19 is only dangerous for the elderly population” (Yes = 0; No = 1). Only the first item was included in regression analyses given similarity between these two perceived risk items.

#### Health concerns

COVID-19 related concerns were assessed by asking “How true are the following statements about COVID-19 for you right now?” for 2 items: “I am worried about my health” and “I am worried about the health of my family members”. Responses to a 5-point Likert scale were categorized as True, False, and Neutral/I don’t know.

#### Sociodemographic measures

Student-reported sociodemographic items included sex/gender (male; female; “I describe my gender differently”/”I prefer not to say” [collapsed into one category due to small sample size]), grade (Grade 9 to 12 in Ontario, Alberta, and BC; Secondary I to V in Quebec, classified as equivalent to Grade 7/8 to 11), and race/ancestry (White; nonwhite [including response options Asian, Black, Latin American/Hispanic, mixed (selected more than one response option), ‘other’, and Métis, First Nations, or Inuit]). Socio-economic status (SES) was assessed by creating a sum score from four items: “In your house, do you have your own bedroom?” (Yes=1; No=0); “Do you sometimes go to bed hungry because there is not enough money to buy food?” (Yes=0; No=1); “Would you say that you and your family are more or less financially comfortable than the average student in your class?” (More comfortable=2; As comfortable-1; Less comfortable=0); and “I am worried about my family being able to pay bills and expenses” (True/Mostly True=0; Neutral/I don’t know/Mostly False/False=1). SES scores have a possible range of 0-5, with higher scores indicative of higher SES. Province was also included given differences in COVID-19 responses.

### Statistical analysis

Frequency statistics were calculated for sociodemographic characteristics, and for the COVID-19 related measures by the two mask support items. SPSS Mixed for Generalized Linear Mixed Models was used with a random intercept at the school level and specifying multinomial distribution with logit link function. Two models were conducted to examine the dependent variables (support for mask use in public spaces and school mask requirements) in association with COVID-19 related prevention discussions, knowledge, perceived risk, and health concerns, controlling for student sociodemographic characteristics (sex/gender, grade, race, SES, province). Descriptive statistics are reported for the full sample and in males and females separately. Regressions models are reported in the full sample only, given corresponding results between females and males. Results for students that reported describing their gender differently or who preferred not to report their gender are not reported separate from the full sample due to small sample size. All analyses were performed in IBM SPSS Version 28.0.

## Results

Table 1 presents the sample sociodemographic characteristics. Most survey participants lived in Quebec (61.8%) or Ontario (25.2%) and identified as white (76.5%). Over half of students identified as female (52.6%); 3.5% indicated that they describe their gender differently than male/female or prefer not to say. Most students reported having their own bedroom (93.7%), being as financially comfortable as their peers (67.6%) and did not go to bed hungry at night (98.2%); 18.2% reported concerns about their family being able to pay bills.

**Table 1:** Sample sociodemographic characteristics

		Total *N* = 42,767 N (%)	Females *N* = 22,660 N (%)	Males *N* = 18,636 N (%)
Province	AB	1823 (4.3)	923 (4.1)	809 (4.3)
	BC	3716 (8.7)	1967 (8.7)	1531 (8.2)
	ON	10,779 (25.2)	5775 (25.5)	4585 (24.6)
	QC	26,449 (61.8)	13,995 (61.8)	11,711 (62.8)
Grade	Sec I/II in QC	12,127 (28.4)	6167 (27.2)	5626 (30.2)
	9	9364 (21.9)	4879 (21.5)	4091 (22.0)
	10	9719 (22.7)	5294 (23.4)	4103 (22.0)
	11	8013 (18.7)	4405 (19.4)	3322 (17.8)
	12	3544 (8.3)	1915 (8.5)	1494 (8.0)
Race	White	32,707 (76.5)	17,431 (76.9)	14,375 (77.1)
	Nonwhite	10,060 (23.5)	5229 (23.1)	4261 (22.9)
Own bedroom	Yes	40,073 (93.7)	21,236 (93.7)	17,506 (93.9)
	No	2694 (6.3)	1424 (6.3)	1130 (6.1)
Hungry at bedtime	Yes	771 (1.8)	383 (1.7)	292 (1.6)
	No	41,996 (98.2)	22,277 (98.3)	18,344 (98.4)
Relative Family Affluence	More	10,866 (25.4)	5133 (22.7)	5458)29.3)
	As comfortable	28,901 (67.6)	15,857 (70.0)	12,053 (64.7)
	Less	3000 (7.0)	1670 (7.4)	1125 (6.0)
Concern about family paying bills	True	7795 (18.2)	4441 (19.6)	2960 (15.9)
	Neutral/False	34,972 (81.8)	18,219 (80.4)	15,676 (84.1)
		**M (SD)**		
SES score	Continuous (0–5)	3.92 (0.83)	3.88 (0.83)	4.00 (0.80)

Survey data from Canadian secondary school students that participated in the COMPASS study during the 2020/21 school year

See Table 2 for frequency of support mask wearing in indoor public spaces and for school requirements to wear masks, and Tables 3 and 4 for frequency statistics across support for mask wearing in public spaces and school mask requirements by the COVID-19 related items, respectively. In the full sample, most students supported wearing masks in indoor public spaces (81.9%) and school requirements for students to wear masks (67.8%); about 9% of students were unsupportive for mask use in each category. A greater proportion of females supported wearing masks in indoor public spaces (83.9% vs. 79.1%) and school mask requirements (70.8% vs. 63.5%) than males. Among students who were supportive of their school requiring masks, 94.7% were also supportive of wearing masks in public spaces (64.2% of total sample). Only 55.7% of students who were unsupportive of schools requiring masks were also unsupportive of wearing masks in public spaces (5.1% of total sample). The majority (66.8%) of students who were neutral regarding mask requirements in schools reported support for mask wearing in indoor public spaces (15.4% of sample), while students who were neutral or undecided about wearing masks in indoor public spaces tended to also be neutral about school mask requirements (57.7%; 5.4% of sample).

**Table 2:** Support for wearing masks in indoor public spaces by support for schools requiring mask use

		Mask wearing in indoor public spaces			
		Supportive N (%)	Neutral N (%)	Unsupportive N (%)	Total N (%)
**Full Sample**					
Support for school mask requirements	Supportive	27,453 (64.2)	965 (2.3)	572 (1.3)	28,990 (67.8)
	Neutral	6602 (15.4)	2319 (5.4)	969 (2.3)	9890 (23.1)
	Unsupportive	983 (2.3)	738 (1.7)	2166 (5.1)	3887 (9.1)
	Total	35,038 (81.9)	4022 (9.4)	3707 (8.7)	42,767 (100.0)
**Females**					
Support for school mask requirements	Supportive	15,352 (67.7)	472 (2.1)	229 (1.0)	16,053 (70.8)
	Neutral	3260 (14.4)	1185 (5.2)	461 (2.0)	4906 (21.7)
	Unsupportive	411 (1.8)	376 (1.7)	914 (4.0)	1701 (7.5)
	Total	19,023 (83.9)	2033 (9.0)	1604 (7.1)	22,660 (100.0)
**Males**					
Support for school mask requirements	Supportive	11,056 (59.3)	464 (2.5)	540 (2.9)	11,841 (63.5)
	Neutral	3143 (16.9)	1079 (5.8)	343 (1.8)	4711 (25.3)
	Unsupportive	540 (2.9)	343 (1.8)	1201 (6.4)	2084 (11.2)
	Total	14,739 (79.1)	1886 (10.1)	2011 (10.8)	18,636 (100.0)

Survey data from Canadian secondary school students that participated in the COMPASS study during the 2020/21 school year

**Table 3:** COVID-19 concerns, perceived threat, guideline adherence, discussions, and knowledge by support for wearing masks in indoor public spaces

		Total N (%)	Supportive N (%)	Neutral N (%)	Unsupportive N (%)
**Health concerns**					
Worried about my health	True	8060 (18.8)	7370 (21.0)	408 (10.1)	282 (7.6)
	Neutral	9107 (21.3)	7630 (21.8)	1052 (26.2)	425 (11.5)
	False	25,600 (59.9)	20,038 (57.2)	2562 (63.7)	3000 (80.9)
Worried about family’s health	True	24,752 (57.9)	21,820 (62.3)	1666 (41.4)	1266 (34.2)
	Neutral	8777 (20.5)	6741 (19.2)	1270 (31.6)	766 (20.7)
	False	9238 (21.6)	6477 (18.5)	1086 (27.0)	1675 (45.2)
Little risk to young people	True	16,427 (38.4)	12,715 (36.3)	1672 (41.6)	2040 (55.0)
	Neutral	12,186 (28.5)	10,159 (29.0)	1465 (36.4)	562 (15.2)
	False	14,154 (33.1)	12,164 (34.7)	885 (22.0)	1105 (29.8)
Only dangerous for older adults	Yes	7407 (17.3)	4758 (13.6)	1033 (25.7)	1616 (43.6)
	No	35,360 (82.7)	30,280 (86.4)	2989 (74.3)	2091 (56.4)
**Knowledge**					
Symptoms always present	Yes	3653 (8.5)	2560 (7.3)	520 (12.9)	573 (15.5)
	No	3653 (91.5)	32,478 (92.7)	3502 (87.1)	3134 (84.5)
Masks prevent transmission	Yes	36,614 (85.6)	31,783 (90.7)	2948 (73.3)	1883 (50.8)
	No	6153 (14.4)	3255 (9.3)	1074 (26.7)	1824 (49.2)
Prevention discussions	Always/Sometimes	30,632 (71.6)	26,421 (75.4)	2515 (62.5)	1696 (45.8)
	Never	12,135 (28.4)	8617 (24.6)	1507 (37.5)	2011 (54.2)

Survey data from Canadian secondary school students that participated in the COMPASS study during the 2020/21 school year. Percentages represent proportions of the column n

**Table 4:** COVID-19 concerns, perceived threat, guideline adherence, discussions, and knowledge by support for school mask requirements

		Total N (%)	Supportive N (%)	Neutral N (%)	Unsupportive N (%)
**Health concerns**					
Worried about my health	True	8060 (18.8)	6486 (22.4)	1202 (12.2)	372 (9.6)
	Neutral	9107 (21.3)	6545 (22.6)	2125 (21.5)	437 (11.2)
	False	25,600 (59.9)	15,959 (55.1)	6563 (66.4)	3078 (79.2)
Worried about family’s health	True	24,752 (57.9)	18,552 (64.0)	4772 (48.3)	1428 (36.7)
	Neutral	8777 (20.5)	5352 (18.5)	2591 (26.2)	834 (21.5)
	False	9238 (21.6)	5086 (17.5)	2527 (25.6)	1625 (41.8)
**Perceived risk**					
Little risk to young people	True	16,427 (38.4)	9725 (33.5)	4358 (44.1)	2344 (60.3)
	Neutral	12,186 (28.5)	8541 (29.5)	3006 (30.4)	639 (16.4)
	False	14,154 (33.1)	10,724 (37.0)	2526 (25.5)	904 (23.3)
Only dangerous for elderly	Yes	7407 (17.3)	3664 (12.6)	2117 (21.4)	1626 (41.8)
	No	35,360 (82.7)	25,326 (87.4)	7773 (78.6)	2261 (58.2)
**Knowledge**					
Symptoms always present	Yes	3653 (8.5)	2071 (7.1)	1033 (10.4)	549 (14.1)
	No	3653 (91.5)	26,919 (92.9)	8857 (89.6)	3338 (85.9)
Masks prevent transmission	Yes	36,614 (85.6)	26,554 (91.6)	7876 (79.6)	2184 (56.2)
	No	6153 (14.4)	2436 (8.4)	2014 (20.4)	1703 (43.8)
Prevention discussions	Always/Sometimes	30,632 (71.6)	22,485 (77.6)	6356 (64.3)	1791 (46.1)
	Never	12,135 (28.4)	6505 (22.4)	3534 (35.7)	2096 (53.9)

Survey data from Canadian secondary school students that participated in the COMPASS study during the 2020/21 school year. Percentages represent proportions of the column n

Table 5 presents the results for the two regression models. Adolescents had increased odds of supporting mask use in indoor public spaces if they reported concerns about their own health (AOR 1.99, 95% CI 1.72, 2.31) or their family’s health (AOR 2.42, 95% CI 2.20, 2.67), had discussions regarding ways to prevent infection (AOR 2.22, 95% CI 2.05, 2.40), perceived COVID-19 to be a risk to young people (AOR 1.20, 95% CI 1.09, 1.31), knew that signs are not always present in COVID-19 cases (AOR 2.12, 95% CI 1.89, 2.38), and that masks prevent COVID-19 transmission when someone coughs (AOR 8.25, 95% CI 7.60, 8.96). Likewise, adolescents were more likely to support school mask requirements if they reported concerns about their own health (AOR 1.51, 95% CI 1.32, 1.73) or their family’s health (AOR 2.04, 95% CI 1.86, 2.24), had discussions regarding ways to prevent infection (AOR 2.51, 95% CI 2.32, 2.71), perceived COVID-19 to be a risk to young people (AOR 2.18, 95% CI 1.99, 2.39), knew that signs are not always present in COVID-19 cases (AOR 1.92, 95% CI 1.71, 2.15), and that masks prevent COVID-19 transmission when someone coughs (AOR 7.56, 95% CI 6.94, 8.23).

**Table 5:** Student support for mask use regressed on COVID-19 health concerns, guideline compliance, discussions, and knowledge

**School mask requirements for students**					
***Reference: Unsupportive***		**Neutral**		**Supportive**	
		**AOR**	**95% CI**	**AOR**	**95% CI**
Concerns re: own health (Ref: False)	True	1.09	0.95, 1.26	1.51	1.32, 1.73
	Neutral	1.51	1.34, 1.71	1.43	1.27, 1.61
Concerns re: family’s health (Ref: False)	True	1.49	1.35, 1.64	2.04	1.86, 2.24
	Neutral	1.53	1.37, 1.70	1.42	1.28, 1.58
Little risk to young people (Ref: True)	False	1.36	1.23, 1.90	2.18	1.99, 2.39
	Neutral	1.97	1.77, 2.18	2.16	1.96, 2.39
Prevention discussions (Ref: Never)	Always/Sometimes	1.71	1.58, 1.85	2.51	2.32, 2.71
Masks prevent transmission (Ref: No)	Yes	2.96	2.72, 3.23	7.56	6.94, 8.23
Symptoms always present (Ref: Yes)	No	1.39	1.23, 1.56	1.92	1.71, 2.15
**Mask wearing in indoor public spaces**					
***Reference: Unsupportive***		**Neutral**		**Supportive**	
		**AOR**	**95% CI**	**AOR**	**95% CI**
Concerns re: own health (Ref: False)	True	1.50	1.25, 1.79	1.99	1.72, 2.31
	Neutral	1.98	1.73, 2.27	1.36	1.20, 1.53
Concerns re: family’s health (Ref: False)	True	1.32	1.17, 1.49	2.42	2.20, 2.67
	Neutral	1.81	1.60, 2.05	1.66	1.49, 1.84
Little risk to young people (Ref: True)	False	0.83	0.74, 0.93	1.20	1.09, 1.31
	Neutral	2.38	2.11, 2.69	1.88	1.69, 2.09
Prevention discussions (Ref: Never)	Always/Sometimes	1.54	1.40, 1.70	2.22	2.05, 2.40
Masks prevent transmission (Ref: No)	Yes	2.56	2.32, 2.83	8.25	7.60, 8.96
Symptoms always present (Ref: Yes)	No	1.22	1.07, 1.40	2.12	1.89, 2.38

Survey data from Canadian secondary school students that participated in the COMPASS study during the 2020/21 school year. *AOR* Adjusted odds ratio. Models adjusted for student sex/gender, race, grade, socioeconomic status, province, and school clustering

## Discussion

We examined support of mask use as a public health prevention measure used to control the spread of SARS-CoV-2 transmission among a large sample of Canadian secondary school students during the 2020/21 school year, the first complete school year following the beginning of the pandemic. The majority of secondary school students were supportive of wearing a mask in indoor public spaces and of schools requiring mask use while at school. Factors associated with support were comparable across outcomes in that support for mask use and school requirements were both higher in students that reported higher perceptions of COVID-19 severity and susceptibility for young people, concern about their own or their family’s health, having had discussions around ways to prevent infection, and knowing that individuals with COVID-19 do not always show signs and symptoms, and, in particular, that masks reduce the risk of SARS-CoV-2 transmission when individuals cough.

A higher proportion of adolescents reported being supportive of mask use in indoor public spaces relative to school requirements for student mask use; about 9% were unsupportive across both items, while more students reported being neutral or unsure regarding support for school requirements for mask use than wearing mask in public spaces. The difference may reflect the specification of *mandated* use in the school item, while the public spaces item simply refers to mask wearing. Alternatively, the familiarity and shared social identity in the school context as opposed to other indoor public spaces could play a role. Shared group membership is shown to attenuate health risk perceptions, and in turn, increase health risk behaviours, whereas individuals more readily distance themselves from strangers [30]. It is also possible that concerns about social norms, appearance, comfort, and communication may be more salient in the school context than other public spaces, given the amount of time spent there and as a key context for peer socialization among young people.

Support for masks was higher in females than males, consistent with evidence in adults [17]. The predictors examined in the current study, including COVID-19 related health concerns, knowledge, and discussions, demonstrated similar associations with mask support across sex. Further research is needed to examine factors that may influence varying support for public health measures in population subgroups, including intersections of gender, SES, race, and geographic location, given disparities in access to information, and real and perceived capacity to exercise self determination.

In the first full school year following the onset of the pandemic, most students appear knowledgeable around risks related to COVID-19, transmission, and preventative measures; however, results support a continued need for improved public health messaging and education targeting adolescents. Over one-quarter of students had never had discussions around measures to prevent infection with family, friends, and/or health care professionals, at least 6 months into the COVID-19 pandemic, and these students were less likely to support mask use. Similarly, in previous analyses using COMPASS study data from Quebec adolescents during the first few months of the pandemic, compliance with COVID-19 preventative measures was positively associated with discussions about preventative measures and what to do in case of infection, as well as pandemic-related knowledge and perception of risk related to COVID-19 [23]. Having these discussions during low stress times is advisable; in a longitudinal study of adolescents, more frequent parent-adolescent conversations early in the pandemic predicted increased adherence to preventative behaviours throughout the pandemic when adolescents reported low stress, but when adolescents were high in stress, they predicted decreased adherence via reduced empathetic concern [31].

Improving knowledge about mask use efficacy appears particularly important for adolescent support for mask use and a valuable target for public health messaging. Adolescents that acknowledged that mask use can reduce droplet transmission when a person coughs were 7–8 times more likely to report mask support. Supporting adolescents’ health literacy through discussions with key individuals in their lives appears beneficial and may help combat the overabundance of false and correct information available online [32]. Improving knowledge about mask use efficacy appears particularly important for adolescent support for mask use, and a valuable target for public health messaging. As a dominant source of health information, particularly for younger persons [20], public health agencies have begun to harness social media platforms to disseminate preventative messages [5]. Peers are also likely to be worthwhile targets for messages targeting adolescents. In US youth, the desire to avoid contracting and spreading SARS-CoV-2 and peer influence were associated with mask use [8]. Similarly, in an online survey of Canadian adolescents, social responsibility was associated with more adherence to public health measures while social concerns regarding maintaining social ties and social judgement were associated with less adherence [7].

Results resemble available evidence among adults. Surveys conducted early in the pandemic (July 2020) among Canadian adults found high levels of support for mandatory mask policies in public spaces (74%) [33]. As restrictions were lifting, in March 2022, 73% of Canadians reported that they would support the continuation of mask requirements in public spaces [10]. Adults with positive perceptions of mask use were more likely to report fears of contracting COVID-19; negative perceptions of mask use were associated with discomfort, concerns about appearance and negative attention from others, difficulty establishing the habit, and beliefs that masks are ineffective, possibly harmful, unnecessary (e.g., in the context other protective measures), and an infringement on independence [6, 34, 35]. In adults, resistance to mask use has been explained as psychological reactance, described as a reaction to fear when there is a lack of self-efficacy to reduce the threat [6, 36]. According to the EPPM, when individual’s threat (perceived severity and susceptibility) and efficacy (response efficacy and self-efficacy) appraisals are high, they will be motivated to engage in the adaptative behaviour, but when their threat appraisal is high and efficacy appraisal is low, individuals may enter defensive avoidance to control the fear. Suggestions to improve messaging, and avoid psychological reactance, have included supporting self-efficacy in engaging in preventative behaviours, gain-framed messaging around personal benefits, prosocial framing (i.e., emphasizing social responsibility in terms of how individual choice impacts others), and indirect or subtle “nudges” [37]. Further research is required to test these strategies among adolescents.

### Limitations

Students not responding to the online survey may differ in their perceptions of mask use and the COVID-19 pandemic. Random factors likely contributed to the lower participation of the online survey during the pandemic relative to previous years of the COMPASS study, such as whether schools and classrooms administered the survey during class time. Also, COMPASS is an ongoing general health survey, that added COVID-19 items; potential bias in participant COVID-19 perceptions may be less likely, as a non-COVID-19 specific study. Further, the COMPASS study does not use student names to help improve perceptions of anonymity and the passive consent protocols are shown to be important for collecting robust data by helping to improve response rates and the generalizability of results [38]. However, COMPASS was not designed to be representative. No data were available on potentially important influences on adolescents’ perspectives (e.g., social norms and peer influence) based on developmental stage and evidence from research on mask use policy compliance. Adolescents’ perceptions of support for mask use and policies among their peers and parent(s)/guardian(s) should be considered in future research. Lastly, the COVID-19 pandemic context has changed rapidly, and these data were collected during the 2020/2021 school year, before the omicron variant emerged and coinciding with the original and alpha variant waves in Canada.

## Conclusions

The majority of adolescents in this large population-based sample of secondary school students reported support for mask use in indoor public spaces, as well as school mask requirements, during the 2020/2021 school year. Support for mask use was associated with health concerns, perceptions and knowledge of COVID-19 risk and mask effectiveness, and having had discussions regarding preventing infection. Increasing knowledge around the effectiveness of facial masks appears likely to have the largest impact on mask use support.

## Data Availability

COMPASS study data is available upon request through completion and approval of an online form: https://uwaterloo.ca/compass-system/information-researchers/data-usage-application The datasets used during the current study are available from the corresponding author on reasonable request.

## Abbreviations

BC: British Columbia
COMPASS: Cannabis, Obesity, Mental health, Physical activity, Alcohol, Smoking, and Sedentary behaviour study
COVID-19: Coronavirus disease 2019
EPPM: Extended Parallel Process Model
SARS-CoV-2: Infectious severe acute respiratory syndrome coronavirus-2
SES: Socio-economic status
